# Twitter Followers of Canadian Political and Health Authorities during the COVID-19 Pandemic: What Are Their Activity and Interests?

**DOI:** 10.1017/S0008423921000020

**Published:** 2021-01-14

**Authors:** Michael Haman

**Affiliations:** University of Hradec Králové, Philosophical Faculty, Rokitanského 62, 500 03 Hradec Králové, Czech Republic

**Keywords:** COVID-19, social media, social networks, Twitter, COVID-19, médias sociaux, réseaux sociaux, intégration de Twitter

## Abstract

I examined the use of Twitter during the COVID-19 pandemic to find out how many Twitter users started to follow relevant Canadian political and health authorities, and I investigated their activity and interests. To this end, I analyzed 398,037 Twitter accounts. The results reveal that the Twitter accounts of relevant authorities gained a significant number of new Twitter followers during the pandemic. The Twitter users who joined during the pandemic were rather passive; they tweeted and liked fewer tweets than Twitter users who registered in the months prior to the pandemic. They also chose to follow Twitter accounts predominantly related to news, politics and governmental agencies. These findings suggest that during the pandemic, numerous information-seeking citizens joined Twitter for the purpose of obtaining information about public health matters, which in turn suggests that authorities should incorporate Twitter into their information dissemination tools, especially during emergencies, to meet the public demand for information.

## Résumé

J'ai examiné l'utilisation de Twitter pendant la pandémie COVID-19 pour savoir combien d'utilisateurs ont commencé à suivre les avis des autorités politiques et sanitaires canadiennes compétentes, et j'ai enquêté sur l'activité des suiveurs. À cette fin, j'ai analysé 398 037 comptes Twitter. Les résultats révèlent que pendant la crise sanitaire les comptes Twitter des autorités ont gagné un nombre important de nouveaux adeptes. En outre, les nouveaux utilisateurs de Twitter étaient plutôt passifs; ils ont clavardé et aimé moins de gazouillis que les utilisateurs de la plate-forme qui se sont inscrits dans les mois précédant la pandémie. De plus, ils ont choisi de préférence les comptes Twitter liés à l'actualité, à la politique et aux agences gouvernementales. Ces constatations amènent à penser que de nombreux citoyens ont rejoint Twitter au cours de la pandémie dans le but d'obtenir des informations sur les enjeux de santé publique. Cela suggère que les autorités devraient intégrer Twitter parmi leurs outils de diffusion de l'information, notamment dans des situations d'urgence, pour répondre aux besoins d'information du public.

## Introduction

The 2019 novel coronavirus disease (COVID-19) has significantly affected the lives of people across the world, and Canada has been no exception. Government officials charged with taking measures to prevent the spread of COVID-19 have had several options for informing the public about decisions being made and steps being implemented. They could use traditional media, such as television, radio or print, but they also could use newer forms, such as social media. Social media can be an ideal tool to offer information about the state of the country in times of crisis, when it is crucial to swiftly provide correct information to citizens. One of the social networking services currently quite popular in Canada is Twitter. At the beginning of 2020, almost 7 million Canadians were registered on Twitter (Statista, 2020). For this study, I analyzed the Twitter activity of users who interacted with Canadian authorities on Twitter during the COVID-19 pandemic. Analysis of these interactions is important since there is evidence that the COVID-19 pandemic had an impact on government support (Bol et al., 2020). The Canadian political science community wasted no time in analyzing the COVID-19 pandemic, and a number of excellent studies have been published (Sevi et al., 2020; Malloy, 2020; Pickup et al., 2020; Miller, 2020a, 2020b; van der Linden and Savoie, 2020; Motta et al., 2020). All of these studies have emphasized the importance of the COVID-19 pandemic to the Canadian political system and society. This research note contributes to this collection.

The literature on the uses of social media during crisis events, natural disasters and emergencies clearly shows that governments and their agencies provide information via social media that facilitate managing these situations (Zhang et al., 2019; Tang et al., 2015; Alexander, 2014; Procter et al., 2013; Kavanaugh et al., 2012; Graham et al., 2015). One could assume that governments and their agencies would behave similarly during the COVID-19 pandemic. In recent years, there has also been increased interest in research on political communication via Twitter (Jungherr, 2015, 2016; Dang-Xuan et al., 2013; Enli and Skogerbø, 2013), and the past year has seen the publication of numerous studies on Twitter and user activity during the COVID-19 pandemic.

It is possible to identify three themes emerging from the research on Twitter and COVID-19. The first is the importance of Twitter use by politicians, state leaders and public authorities during the pandemic. Grossman et al. (2020) show the importance of US governors’ recommendations for residents to stay at home, which significantly reduced the mobility of individuals during the pandemic. Rufai and Bunce (2020) analyze the G7 world leaders’ use of Twitter during the COVID-19 pandemic and suggest that Twitter can be used as a platform for rapid communication with citizens on public health matters. In general, many world leaders have already adopted Twitter as a platform for communication with citizens (Barberá and Zeitzoff, 2018). In Canada, specifically, Teichmann et al. (2020) show the importance of Canada's local and provincial authorities use of social media during the COVID-19 crisis.

The second research theme is political polarization on Twitter. Merkley et al. (“A Rare Moment,” 2020) show that in Canada, the political elites and the public have been in a period of cross-partisan consensus on important issues (such as social distancing) during the COVID-19 pandemic; this has notably not been the case in the United States, where tweets have been characterized by strong political polarization (Jiang et al., 2020).

The third theme is the spread of misinformation about COVID-19 on Twitter (Pérez-Dasilva et al., 2020; Rodríguez et al. 2020). In Canada, Bridgman et al. (2020) find a link between the dissemination of misinformation on social media and behaviours and attitudes that complicate managing the COVID-19 pandemic. Pulido et al. (2020) show that even though false information about the pandemic is tweeted more than information based on scientific knowledge, it is retweeted less than science-based tweets. The spread of misinformation is very dangerous in times of crisis, as it can lead to citizens not complying with state regulations and following experts. Several factors affect whether a citizen will be willing to listen to experts. In a Canadian study based on survey experiments, Merkley et al. (“Anti-Intellectualism,” 2020) emphasize the role of anti-intellectualism as an important factor in shaping COVID-19 information.

This research note attempts to fill some of the gaps in the literature mentioned above. For example, previous studies have not examined the impact of the pandemic on the Twitter user base, the increase in the number of Twitter users who follow accounts of relevant authorities, or the behaviour and activity of new Twitter users. It is important to investigate these aspects because they highlight to what degree it is important to use social media during times of crisis. This research note also examines how much potential Twitter has as an information tool for people who do not necessarily consider social media as a typical platform for their entertainment or active communication but who are, instead, interested in using it to obtain information in times of crisis. The third research theme, on the dissemination of misinformation, is particularly relevant, as this study provides a counterpoint, focusing on the degree to which Twitter can be used as an important and legitimate source of information by political actors and health authorities.

There are a number of possible motives behind Twitter use. Parmelee and Bichard (2012) mention, for example, convenience, entertainment, self-expression, guidance, information-seeking and social utility. People who are interested in getting information about COVID-19 may be influenced by several of these. Twitter is a convenient way to quickly obtain information from relevant authorities, and this information can guide people's decisions, such as whether and when it is necessary to wear a face mask. By obtaining information via Twitter, people are informed about government actions and are able to both express opinions and critique perceptions of government activities. Information obtained via Twitter has a social utility that assists followers in their social interaction with friends and family. Numerous studies have also concluded that there are some people who use Twitter primarily as an information-seeking platform (Hughes et al., 2012; Johnson and Yang, 2009).

I have divided this research note into several sections. In the first, I detail the data collection process. In the second, I present the content of tweets from analyzed Twitter accounts via word clouds, in order to examine the communication of officials about COVID-19. In the third, I offer the results of the analysis regarding the growth of Twitter followers: I identify the state of followers before and during the COVID-19 pandemic, and I show the effect of the COVID-19 pandemic on the number of people following Twitter. In the last three sections, I build on the results of the previous sections by presenting findings on people who joined Twitter at the beginning of the pandemic and analyzing their activity and interests on the social media platform.

### Data Collection

For my analysis, I focused on the Canadian government and its officials and agencies. Initially, I collected tweets by Prime Minister Justin Trudeau (@JustinTrudeau) to generate word clouds and basic information about his followers. Next, I gathered detailed information about the Twitter followers of Health Canada and the Public Health Agency of Canada (PHAC). Health Canada and the PHAC have a joint Twitter account: @GovCanHealth. Trudeau, as a leading Canadian politician, is charged with informing the public in general terms, while Health Canada and the PHAC, as a governmental department and agency responsible for crafting federal public health policy, are responsible for publishing updates and timely information about the COVID-19 pandemic for the public. I then analyzed two other Twitter accounts related to the government and public health that were significantly active during the COVID-19 pandemic: the Twitter account of Patty Hajdu, the Minister of Health (@PattyHajdu), and the Twitter account of Theresa Tam (@CPHO_Canada), the Chief Public Health Officer and head of the PHAC Canada. I collected data through the Twitter application programming interface (API) from June 15 to June 20, 2020. More details are given below with each figure, and the summary of data is in Table 1 in the online Appendix. However, for more detailed analysis, I also collected data on the Canadian Space Agency (@csa_asc) and Environment and Climate Change Canada (@environmentca).

### Word Clouds

**Figure 1:**
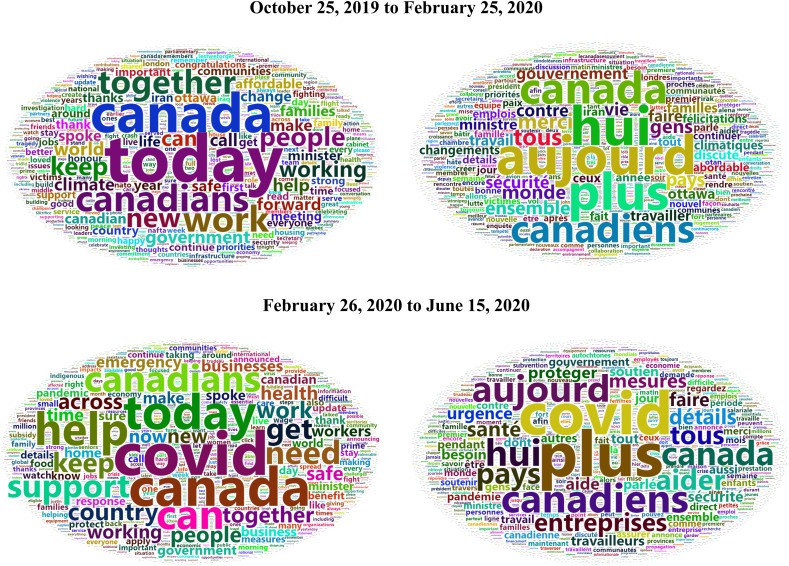
@JustinTrudeau Word Clouds

Figure 1 shows two word clouds based on tweets from @JustinTrudeau. Word clouds are one of the simplest ways to visualize the content of tweets during a specific period. On January 26, 2020, Trudeau first tweeted about COVID-19 by retweeting a tweet by Theresa Tam (@CPHO_Canada). The word clouds in Figure 1 show the words most often tweeted by @JustinTrudeau from January 26 to June 15, 2020. It is important to note that Trudeau posts the same information in separate English and French tweets. Fortunately, Twitter API offers information about the language of tweets. Therefore, I created a separate word cloud for each language.

I excluded stop words from each tweet by using R package stopwords (Benoit et al., 2020) and used snowball as the source of stop words. As I continued processing tweets, I deleted numbers and converted words to lowercase for consistency. The generated word clouds clearly show that the word *COVID* was the most frequently used within English tweets during the examined period, and it was the second most frequently used word in the French tweets (the general French adverb *plus* was the most used). Other frequently used words found in Trudeau's tweets were those connected with morale-boosting related to the pandemic. In English, those words included *Canada*, *Canadians*, *Canadian* or *country*, underscoring the importance of uniting as a nation; other words such as *help*, *support*, *keep*, *together* and *protect* emphasize the need for cooperation and encourage citizens to consider others. For comparison, two word clouds based on tweets that preceded the COVID-19 pandemic are presented in the top half of Figure 1. It is possible to see that some words, such as *Canada* and *Canadians*, were used more frequently before the pandemic but that other words, such as *support*, *help* and *need*, were used more often during the pandemic.

**Figure 2:**
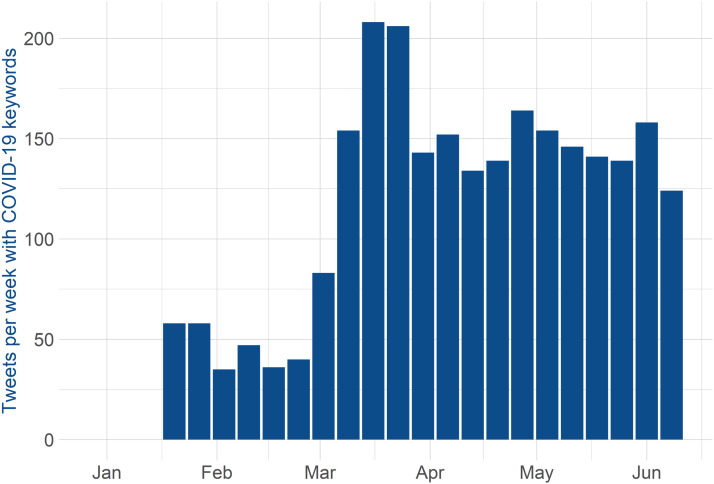
Tweets per Week with COVID-19 Keywords from @JustinTrudeau, @GovCanHealth, @PattyHajdu and @CPHO_Canada

Figure 2 shows the number of tweets per week with COVID-19 keywords (*coronavirus* and *covid*) from all four analyzed accounts. For this analysis, I included only original tweets, since these accounts often retweet to each other. Figure 2 illustrates the over-time dynamics of using Twitter in Canadian government communication. The government had started informing the public since the latter part of January, as more news started coming from China and other parts of the world about the significance of the coronavirus. Also, on January 27, 2020, Canada had their first confirmed COVID-19 case, an individual returning from China (CTV News, 2020). On March 5, 2020, the first case of COVID-19 community transmission in Canada was confirmed (Slaughter, 2020). In March, the number of infections started to increase, which led to more information coming from the government. Figure 2 shows the spike in mid-March; after the spike, the level of information coming from the government was relatively constant.

### The Growth of Twitter Followers

**Figure 3:**
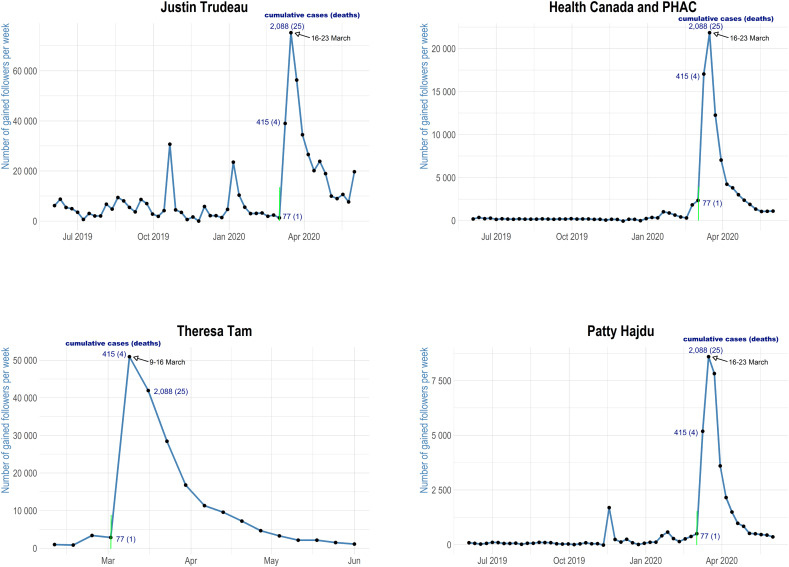
Number of Followers Gained for Each Twitter Account

Information-seeking citizens would be expected to follow Twitter accounts providing information about the COVID-19 pandemic if they were interested in relevant updates. Figure 3 demonstrates the growth of followers in four separate graphs. The graphs show the number of Twitter followers gained by @JustinTrudeau, @GovCanHealth and @PattyHajdu from June 3, 2019 to June 8, 2020, and the number gained by @CPHO_Canada from February 10 to June 8, 2020. Unfortunately, Twitter API does not offer information about the growth of followers. Therefore, I gathered continuous data from June 2019 to June 2020. Also, I do not have data on @CPHO_Canada followers before February 2020.

From each graph, it is possible to compare the pre-pandemic period to a period during the COVID-19 pandemic. As mentioned above, on March 5, 2020 (green line in figures), the first case of COVID-19 community transmission in Canada was confirmed (Slaughter, 2020). Therefore, it is reasonable to expect increased interest in information on COVID-19 starting around that date. The graphs in Figure 3 confirm this expectation, showing a sharp increase in followers among all four accounts. For better illustration, the number of cumulative COVID-19 cases and deaths in Canada is provided for three weeks. The source of data is the Johns Hopkins University School of Medicine (Johns Hopkins Coronavirus Resource Center, 2020). The sharpest increase appears approximately two weeks after the first case of community transmission. In one week, from March 16 to March 23, 2020, @JustinTrudeau gained more than 70,000 followers, @GovCanHealth gained almost 25,000 followers, @CPHO_Canada gained more than 40,000 followers and @PattyHajdu gained more than 8,000 followers. The annotation of weeks gives the dates of March 16 to March 23 rather than March 16 to March 22 because the collection of the number of followers was done over the course of every Monday.

All the Twitter follower gains are significant, but their significance differs because of the total number of followers. Prior to the pandemic, Trudeau's Twitter account had a far greater number of followers than the other accounts; it is not unexpected that he would see a correspondingly large increase. As of June 8, 2020, @JustinTrudeau had more than five million followers, @GovCanHealth had over 321,000 followers, Tam had over 201,000 followers and Hajdu had over 60,000 followers.

**Figure 4:**
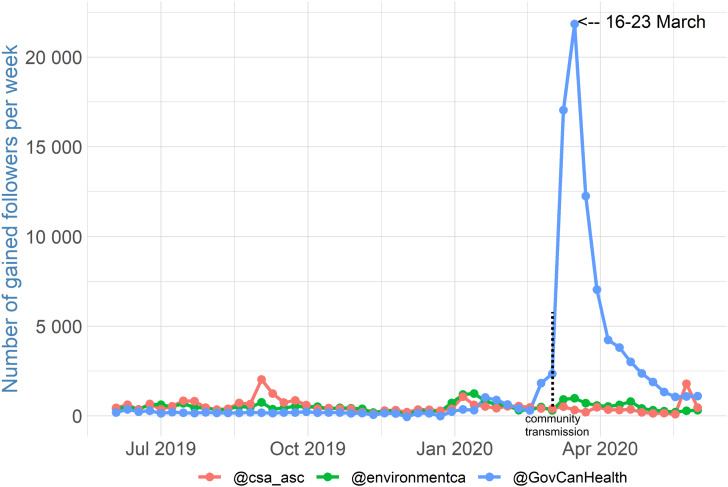
Number of Followers Gained for Twitter Accounts of Different Agencies

However, the significant gains in the number of followers for the analyzed accounts could correspond to a more general trend during the COVID-19 pandemic. Under conditions of anxiety and crisis, people consume more information overall (Gadarian and Albertson, 2014), and indeed, there has been a general increase in information consumption during the COVID-19 pandemic (Casero-Ripolles, 2020). As a placebo test, I plotted the accounts of other departments and agencies to see the difference. I chose the Canadian Space Agency (@csa_asc) and Environment and Climate Change Canada (@environmentca), since they are highly followed on Twitter and not directly related to the COVID-19 pandemic. At the beginning of June 2019, both accounts had a larger following than @GovCanHealth (almost 230,000 followers): @csa_asc had over 260,000 followers, while @environmentca had over 360,000 followers. Figure 4 shows that @csa_asc and @environmentca usually gained slightly more followers than @GovCanHealth in the weeks and months preceding the COVID-19 pandemic. In mid-March, neither @csa_asc nor @environmentca saw the type of spike in followers as seen for @GovCanHealth; Twitter accounts related to the COVID-19 pandemic gained a significant number of followers, while these two did not.

### New Twitter Users

**Figure 5:**
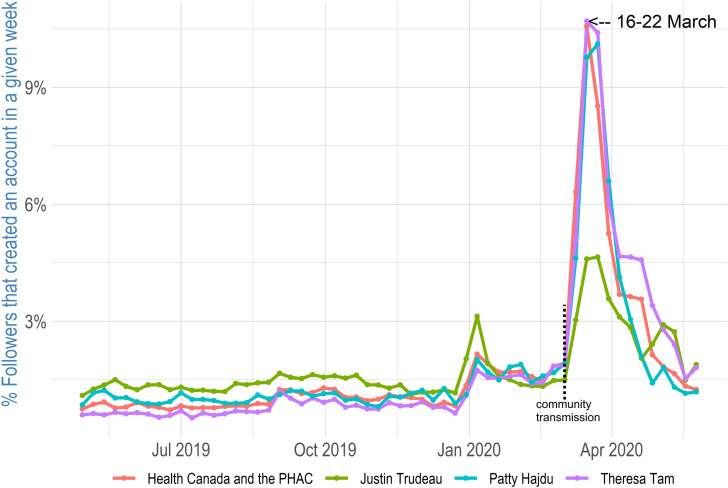
Twitter Followers Who Created an Account in a Given Week

The finding on the number of followers is only one part of the investigation; it is also important to determine how many Twitter followers created new accounts for the purpose of following COVID-19 updates (compared to new followers who already had Twitter accounts). To answer this question, Figure 5 shows the percentage of followers that created a Twitter account each week. Twitter API allows for the collection of the number of followers of each account and offers information about when accounts were created. I looked at all Twitter followers from the four analyzed accounts on June 20, 2020, and I gathered information that allowed me to see when their accounts were created. Therefore, I could split followers into weeks when their accounts were created. However, I calculated the percentage only from people who created Twitter accounts from April 29, 2019, to May 31, 2020. The sum of percentage values from each week is shown on the *x*-axis and equals 100 per cent. The total number of follower accounts that I analyzed included 330,715 for @JustinTrudeau, 25,459 in the case of @GovCanHealth, 32,828 in the case of @CPHO_Canada and 9,035 in the case of @PattyHajdu. Accounts created during the COVID-19 pandemic are, for all analyzed accounts, significantly overrepresented. Twitter users who created accounts between March 16 and March 22, 2020, make up more than 10 per cent of all analyzed accounts following Health Canada and the PHAC, Tam and Hajdu. In the case of Trudeau, the week of March 16 to March 22, 2020, has the largest proportion of created accounts, reaching almost 5 per cent, but it is a smaller percentage when compared to other accounts. The cause of this discrepancy may be that Trudeau had a much larger following already, and he is not as specifically connected to health care topics as the other three accounts. Therefore, while Twitter accounts created in the pre-pandemic period make up around 1.5 per cent or less of analyzed Twitter followers, Twitter accounts created during March and April of 2020 make up significantly more. This implies that the growth of Twitter followers during the COVID-19 pandemic, as described above, is associated with the fact that many of the new followers were from newly created accounts. Therefore, it is possible to conclude that people created new accounts and started using Twitter during the COVID-19 pandemic to receive information from relevant authorities.

### User Activity of New Twitter Followers

**Figure 6:**
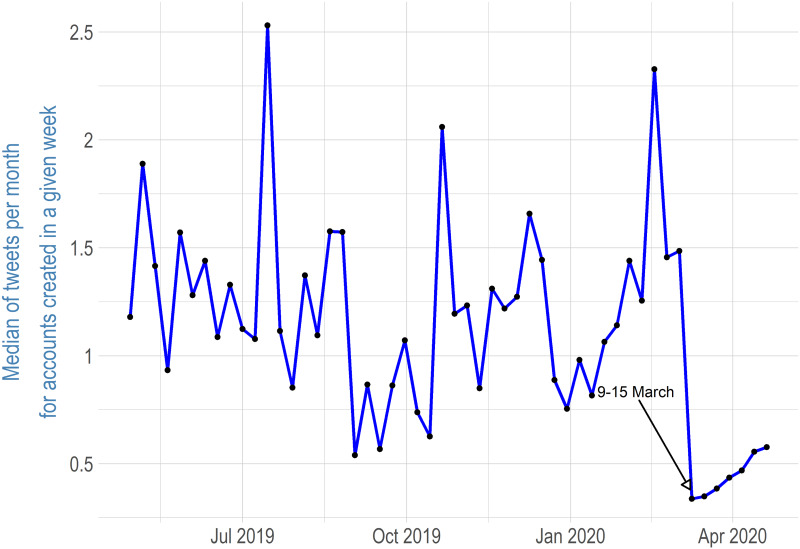
Median of Tweets per Month for Accounts Created Each Week among @GovCanHealth Followers

People who joined Twitter during the COVID-19 pandemic could be expected to have different behaviour on Twitter, since they were primarily information-seeking users and less interested in social interaction; this assumption is based on the fact that they were not sufficiently interested in Twitter before the COVID-19 pandemic to create an account. The analysis of user activity for this part of the study was based on the followers of @GovCanHealth, as it is the Twitter account with the largest number of followers that is strictly related to the Canadian public health system. Figure 6 shows the median number of tweets per month for accounts created each week. For each Twitter account/follower, I divided the total number of tweets by the number of days from the creation of an account to calculate the average number of tweets per day since the Twitter account was created; I then multiplied by 30 to get a number corresponding to one month. Each week is reported separately to account for possible different activity rates depending on how long a user has been active on Twitter. This is also why I included only Twitter accounts created from April 29, 2019, to April 26, 2020, and I excluded accounts created recently, since one could argue that they had not had much time to get acquainted with the social media platform. I examined 23,669 accounts. Figure 6 shows that for accounts created before the COVID-19 pandemic, the median of tweets per month ranged from 0.5 to 2.5. This finding corresponds with the research on Twitter activity in the United States by the Pew Research Center (Hughes and Wojcik, 2019) that found that the median number of tweets per month is 2 in the United States. This number is quite low, as Pew Research Center found that the 10 per cent of users who tweeted the most created 80 per cent of the total number of tweets.

**Figure 7:**
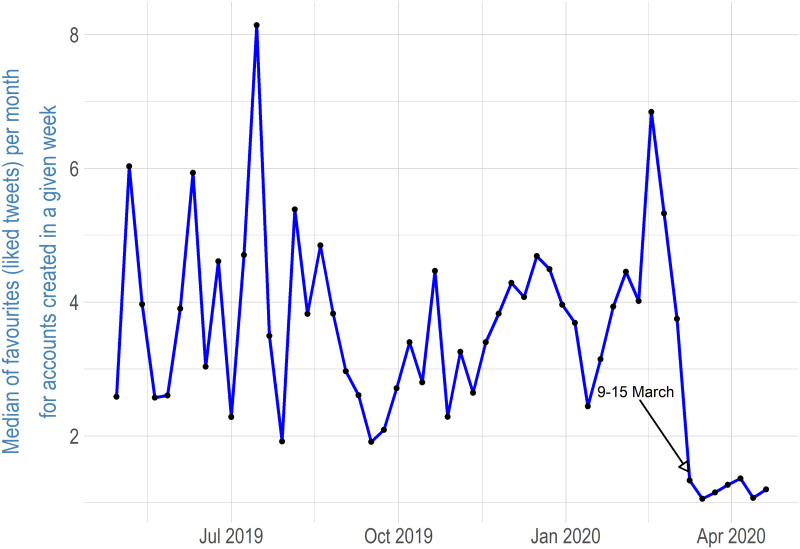
Median of Liked/Favourite Tweets per Month for Accounts Created Each Week among @GovCanHealth Followers

Twitter users who created accounts a few days before and after COVID-19 was declared a pandemic by the World Health Organization (WHO), which happened on March 11, 2020, have significantly fewer tweets per month. For @GovCanHealth followers who created accounts during the week of March 9, 2020, and during several of the weeks following, the median number of tweets is around 0.25. This suggests that users who created a Twitter account during the pandemic period used Twitter mainly as a source of information rather than as a platform for sending tweets. Twitter users can “like” tweets, and it is one type of account interaction; I applied the same process described above for liked tweets and tweets marked as “favourite.” Figure 7 shows a median of liked tweets per month for accounts created each week following @GovCanHealth. The like/favourite results are very similar to the number of tweets. The median of liked/favourite tweets ranges from 2 to 8, depending on the week, for accounts created before the COVID-19 pandemic, while the median of liked/favourite tweets drops to just 1 for accounts created during the COVID-19 pandemic. This implies that @GovCanHealth followers who joined Twitter during the COVID-19 pandemic have significantly different behaviour on Twitter. The limitation of this finding is that it is not possible to find out how many times newly joined Twitter users watch their feed or whether they connect after registration on Twitter at all. Therefore, it is not possible to determine whether passive behaviour was different.

### The Interests of New Twitter Users

**Figure 8:**
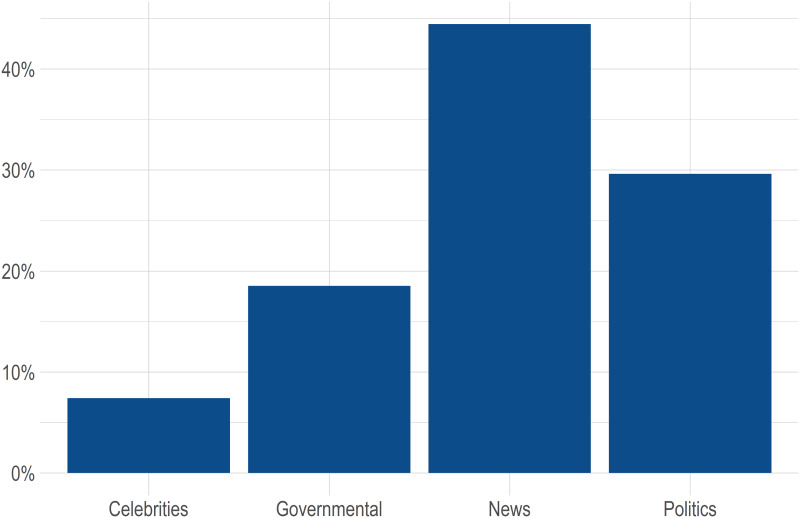
@GovCanHealth Followers Other Most-Followed Twitter Accounts, by Category

To further examine the accounts of people who joined Twitter during the early months of the COVID-19 pandemic, I collected data on the followers of @GovCanHealth and examined what other accounts they followed to look for common interests. I analyzed only the accounts that were created in March and April 2020, and it was only possible to analyze accounts that were not set to private. The total number of examined accounts was 10,698. If, as hypothesized, these new Twitter users are primarily information-seeking citizens interested in information about the COVID-19 situation, then they should follow accounts that are likely to provide them with this information. I classified all Twitter accounts that were followed by at least 10 per cent of followers of @GovCanHealth into four categories: celebrities, governmental, news and politics. Governmental accounts are not necessarily connected with the Canadian government since I included, for example, @WHO and @CDCgov as governmental accounts. A total of 54 Twitter accounts were followed by more than 10 per cent of followers of @GovCanHealth The hypothesis was that newly joined Twitter users would be most interested in news, politics and governmental Twitter accounts rather than celebrities, since celebrities offer different kinds of information than relevant authorities do—although celebrities can, of course, also tweet about COVID-19. Figure 8 is a bar graph that shows the percentage of each account category. All 54 Twitter accounts with detailed information can be found in Table 2 of the Appendix. New followers of @GovCanHealth followed Trudeau's account the most (57 per cent), and 46 per cent of those following @GovCanHealth also followed @CPHO_Canada. From all 54 Twitter accounts, only four celebrities were followed by more than 10 per cent of the followers of @GovCanHealth; all other followed accounts were governmental, news or politics—and news was the dominant category. This indicates that the Twitter users who joined in March and April 2020 were primarily information-seeking users and interested in following updates about the COVID-19 pandemic.

However, it must be emphasized that these findings do not imply that the interests of people who joined Twitter during the COVID-19 pandemic are significantly different from other Twitter users. In comparison to all Canadian Twitter users, it can be expected that @GovCanHealth followers are always skewed toward news-oriented Twitter accounts, regardless of whether they joined Twitter during or before the COVID-19 pandemic. Therefore, these findings cannot help us with the answer to what users who joined Twitter during the COVID-19 pandemic are interested in, compared to other followers of @GovCanHealth, and whether their preferences are systematically different across the board.

## Discussion

The findings in this research suggest that Twitter has served as an information source for many during the COVID-19 pandemic. Several observations support this claim. First, there was a significant increase in people following the Twitter accounts of relevant authorities (Trudeau, Tam, Hajdu, and Health Canada and the PHAC) during the COVID-19 pandemic. Second, for all four Twitter accounts, the number of followers who created accounts at the beginning of the COVID-19 pandemic and in the following weeks is significantly overrepresented when compared to the pre-pandemic months. This means that during that period, new Twitter accounts represented a significant portion of new followers. Although many people were using Twitter before the COVID-19 pandemic, a significant number decided to join at this time. Third, the followers of @GovCanHealth and those who created an account during the COVID-19 pandemic had significantly different behaviour from users who had joined Twitter before the COVID-19 pandemic; these new users posted fewer tweets and liked fewer tweets. This suggests that these new users are more passive and more interested in obtaining information than in interaction. Fourth, I analyzed what other accounts followers of @GovCanHealth followed and determined that they were mainly drawn to Twitter accounts related to news, politics and governmental agencies and, to a much lesser degree, celebrities. This suggests that these new users turned to Twitter primarily as information-seekers rather than entertainment-seekers. All findings suggest that even in countries such as Canada where Twitter is highly popular, there is still room for growth. When people who do not normally use Twitter join it to seek information during a crisis, politicians and state agencies should recognize this behaviour and use social media as one of the platforms for spreading news and information. Also, this research note provides a counterpoint to the literature that emphasizes the role that social media have played in spreading misinformation during the COVID-19 pandemic. Relevant authorities can provide legitimate information to citizens via social media, and the findings show that many citizens are indeed interested in this news.

It is important to note that Twitter is used by a minority of people in Canada and that it is not the dominant source of news information. Only 16 per cent of the Canadian public uses Twitter as a source for political news. The dominant mode of news consumption in Canada is still television (Owen et al., 2020). Therefore, the statements of the relevant authorities from television programs will reach a larger audience. The findings of this study must be considered in this context.

One of the limitations of the study is that it was not possible to determine whether newly gained followers are Canadians and legitimate accounts; however, the same limitation applies to the pre-pandemic months. At the moment, there is no reason to assume that the number of fake accounts would be significantly greater during the COVID-19 pandemic than before, and the more detailed analysis of @GovCanHealth followers did not indicate such an increase. As the pandemic continues, further studies could continue the analysis of Twitter accounts of federal and provincial political actors and authorities, taking the research in different directions.

## Supplementary material

For supplementary material accompanying this paper visit https://doi.org/10.1017/S0008423921000020. click here to view supplementary material
