# Supplementary material for: Twitter Followers of Canadian Political and Health Authorities during the COVID-19 Pandemic: What Are Their Activity and Interests?
Source: Can J Polit Sci. 2021 Jan 14:1–16. doi: 10.1017/S0008423921000020 (PMC7985657; doi:10.1017/S0008423921000020)
Supplement: Supplementary file 1 [file S0008423921000020sup001.docx]

**Appendix**

| Table 1. Data collection | | | |
| --- | --- | --- | --- |
| Data | Analysis/Presentation | Date collection | N |
| @JustinTrudeau tweets | Figure 1 | May 8, 2020 and June 20, 2020 | 3,973^a^ |
| @JustinTrudeau, @GovCanHealth, @PattyHajdu and @CPHO_Canada tweets | Figure 2 | June 20, 2020 | 2,519 ^a^ |
| Twitter followers count | Figure 3 and Figure 4 |  |  |
|  | @GovCanHealth | June 3, 2019 until  June 15, 2020 (each Monday) | 54^b^ |
|  | @PattyHajdu | June 3, 2019 until  June 15, 2020 (each Monday) | 54^b^ |
|  | @JustinTrudeau | June 3, 2019 until  June 15, 2020 (each Monday) | 54^b^ |
|  | @CPHO_Canada | February 10, 2020 until  June 15, 2020 (each Monday) | 18^b^ |
|  | @csa_asc | June 3, 2019 until  June 15, 2020 (each Monday) | 54^b^ |
|  | @environmentca | June 3, 2019 until  June 15, 2020 (each Monday) | 54^b^ |
|  |  |  |  |
| Twitter followers | Figure 5 |  |  |
|  | *Accounts:* |  |  |
|  | @GovCanHealth | June 20, 2020 | 25,459^c^ |
|  | @PattyHajdu | June 20, 2020 | 9,035^c^ |
|  | @JustinTrudeau | June 20, 2020 | 330,715^c^ |
|  | @CPHO_Canada | June 20, 2020 | 32,828^c^ |
|  | Figure 6 and 7 | June 20, 2020 | 23,669^d^ |
|  |  |  |  |
|  | Figure 8 | June 20, 2020 | 10,698^e^ |
| ^a^ tweets; ^b^ weeks; ^c^ only Twitter users/followers that created accounts from April 29, 2019 to May 31, 2020; ^d^ only Twitter users/followers that created accounts from April 29, 2019, to April 26, 2020; ^e^ only Twitter users that created accounts in March and April 2020 | | | |

| Table 2. Twitter accounts that are also followed by @GovCanHealth followers | | | | |
| --- | --- | --- | --- | --- |
| Group | Twitter username | Name | % | Followers |
| Politics | JustinTrudeau | Justin Trudeau | 57.2 | 5.1M |
| News | CBCNews | CBC News | 47.8 | 2.9M |
| Governmental | CPHO_Canada | Dr. Theresa Tam | 46.7 | 203.0K |
| Politics | CanadianPM | CanadianPM | 45.6 | 390.1K |
| News | CTVNews | CTV News | 40.5 | 968.3K |
| Politics | BarackObama | Barack Obama | 38.9 | 120.0M |
| Politics | realDonaldTrump | Donald J. Trump | 37.6 | 82.4M |
| News | globalnews | Globalnews.ca | 36.4 | 508.5K |
| News | CBCAlerts | CBC News Alerts | 33.8 | 1.3M |
| Politics | fordnation | Doug Ford | 32.9 | 315.1K |
| News | CNN | CNN | 31.5 | 48.7M |
| News | globeandmail | The Globe and Mail | 27.5 | 1.8M |
| News | BBCBreaking | BBC Breaking News | 25.5 | 44.3M |
| News | CP24 | CP24 | 24.3 | 1.9M |
| News | nytimes | The New York Times | 23.9 | 46.8M |
| News | CBCTheNational | CBC News: The National | 22.7 | 312.0K |
| Politics | WhiteHouse | The White House | 21.2 | 23.5M |
| Governmental | WHO | World Health Organization (WHO) | 20.9 | 8.0M |
| News | cnnbrk | CNN Breaking News | 20.8 | 58.1M |
| Celebrities | TheEllenShow | Ellen DeGeneres | 20.6 | 80.0M |
| News | TorontoStar | Toronto Star | 20.4 | 1.0M |
| News | nationalpost | National Post | 20.2 | 869.8K |
| News | CBC | CBC | 19.2 | 555.2K |
| News | BBCWorld | BBC News (World) | 18.6 | 28.4M |
| Governmental | Canada | Canada | 17.9 | 791.2K |
| Celebrities | elonmusk | Elon Musk | 17.7 | 36.2M |
| News | CTVToronto | CTV Toronto | 17.3 | 532.0K |
| News | CityNews | CityNews Toronto | 16.3 | 622.0K |
| Politics | HillaryClinton | Hillary Clinton | 14.9 | 28.2M |
| Celebrities | BillGates | Bill Gates | 14.8 | 51.0M |
| Governmental | NASA | NASA | 14.8 | 38.3M |
| Governmental | CanBorder | Canada Border Services Agency | 14.0 | 105.3K |
| News | CBCToronto | CBC Toronto | 14.0 | 572.1K |
| Politics | POTUS | President Trump | 13.5 | 30.6M |
| Governmental | CDCgov | CDC | 13.4 | 2.9M |
| Politics | PattyHajdu | Patty Hajdu | 13.2 | 61.3K |
| Politics | MichelleObama | Michelle Obama | 13.1 | 15.9M |
| News | CBCCanada | CBC Canadian News | 13.1 | 220.0K |
| News | Reuters | Reuters | 12.9 | 22.1M |
| Governmental | ONThealth | ONTHealth | 12.9 | 66.3K |
| Governmental | CanRevAgency | Canada Revenue Agency | 12.9 | 160.3K |
| Governmental | CitImmCanada | IRCC | 12.8 | 267.5K |
| News | weathernetwork | The Weather Network | 12.8 | 1.6M |
| Governmental | Safety_Canada | Public Safety Canada | 12.7 | 116.6K |
| Politics | liberal_party | Liberal Party | 12.5 | 332.4K |
| News | washingtonpost | The Washington Post | 12.2 | 16.0M |
| Celebrities | Oprah | Oprah Winfrey | 11.9 | 43.2M |
| Politics | BernieSanders | Bernie Sanders | 11.6 | 12.4M |
| News | TIME | TIME | 11.3 | 17.3M |
| Politics | theJagmeetSingh | Jagmeet Singh | 11.3 | 313.2K |
| Politics | AndrewScheer | Andrew Scheer | 10.9 | 226.0K |
| Politics | CPC_HQ | Conservative Party | 10.7 | 217.8K |
| Politics | JoeBiden | Joe Biden | 10.2 | 6.4M |
| News | globalnewsto | Global News Toronto | 10.0 | 206.7K |
| N = 10,698 followers of Health Canada and the PHAC that joined Twitter in March or April 2020; % - How many of @GovCanHealth followers in the percentage followed a given account; Followers - How many Twitter followers followed each account | | | | |
